# Preventable hand injuries: A national audit

**DOI:** 10.1016/j.jpra.2023.10.005

**Published:** 2023-10-05

**Authors:** Jennifer C.E. Lane, Anna Loch Wilkinson, Grey Giddins, Maxim D. Horwitz, on behalf of the Hand Injury Prevention Collaborative, William Mason, Alexander Curtis, Fatumata Jalloh, Kudamaduwage Gomes, Rajesh Gopireddy, Niting Sawant, Jemma Rooker, Langhit Kurar, Nik Jagozinski, Andrew Wright, Juliana Hughes, Sayhow Teoh, Alex Nicholls, Rishi Das, Ryan Trickett, Michael David, Haneen Abed, Zaf Naqui, Christiana Lipede, Mo Akhavani, Francis Banhidy, Catrin Wigley, P Caine, Samim Ghorbanian, Sam Gidwani, Charles Bain, Jamil Moledina, Alastair Hunter, John Hardman, Meg Birks, Thomas Goldsmith, Andrej Salibi, Dominic Power, Abdus Burahee, Adrian Murphy, Helen Wohlgemut, Jeremy Rodrigues, Kenaka Bernard, Simon Wimsey, George Matheson, Joshua Ong, Robert Poulter, Segun Ayeko, Sami Hassan, David Warwick, Sherif Fetouh, Charlotte Wray, Mehitab Adel, Onur Berber, Rory Cuthbert, George Wheble, Emily West, Edmund Wright, Lisa Leonard, Emma Reay, Rebecca Martin, David Clarke, Sian Sokota

**Affiliations:** a Barts Bone & Joint Health, Blizard Institute, Queen Mary University of London, 4 Newark Street, E1 2AT, UK; b Nuffield Department of Orthopaedics, Rheumatology and Musculoskeletal Sciences, Nuffield Orthopaedic Centre, University of Oxford, Windmill Road, Oxford, OX3 7LD, UK.; c Chelsea and Westminster Hospital, 369 Fulham Road, SW10 9NH London, UK; d Royal United Hospital, Combe Park, Bath, BA1 3NG, UK

**Keywords:** Injury, Hand, Prevention, National

## Abstract

Little is known of the scale of avoidable injuries presenting to medical services on a national level in the UK. This study aimed to assess the type and incidence of preventable wrist and hand injuries (as defined by the core research team) at a national level in the UK. 28 UK hospitals undertook a service evaluation of all hand trauma cases presenting to their units over a 2 week period in early 2021 identifying demographical and aetiological information about injuries sustained. 1909 patients were included (184 children) with a median age of 40 (IQR 25-59) years. The commonest five types of injury were fractures of the wrist; single phalangeal or metacarpal fractures; fingertip injuries; and infection, with the most common mechanisms being mechanical falls and manual labour. This is the first extensive survey of preventable hand injuries in the UK, identifying a need for further work into prevention to reduce healthcare burden and cost. 50% of injuries presenting to hand surgeons are preventable, with the most common injuries being single fractures of the wrist, phalanx and metacarpal. Few preventable injuries were related to alcohol or narcotic intoxication. Further research is needed to identify how to initiate injury prevention measures for hand injuries, particularly focussed towards hand fracture prevention.

## Introduction

Some hand injuries are avoidable, yet they constitute an acceptable risk of day-to-day living, such as playing sport. To enable further research in this area, a recent definition of preventable or avoidable injuries has been established.^1^ Avoidable hand injury is defined as ‘an injury (e.g. laceration, abrasion) to the hand that is innate to the activity being performed and that would not have occurred if reasonable human interventions were in place’. Silber *et al.* focussed on preventable injuries in a single UK hospital, and tried to establish the magnitude of the problem and whether it was measurable within this one centre. Recent work in the US identified a significant burden of disease associated with hand and finger lacerations, predominantly in men presenting with knife injuries.^2^ Other studies have focussed on specific mechanisms of injury such as injuries to children in school, sports or work-related injury.^3^, ^4^, ^5^, ^6^ In comparison, little is known of the scale of avoidable injuries presenting to medical services on a national level in the UK. Furthermore, work on external validation of the definition of avoidable injury has not been reported. Therefore, a national study to determine both the national burden of avoidable injury and assessing whether the definition of avoidable injury was suitable appeared to be pertinent in this scenario.

### Aims and objectives

The aim of the study was to firstly determine the feasibility of data collection pertaining to preventable wrist and hand injuries at the national level in the UK and secondly to describe the type and incidence of these injuries. The study aimed to highlight the common causes of preventable injuries, to identify key areas potentially amenable to intervention, and to understand the cost to the NHS and burden of injury upon society.

## Methods

### Data source

**Figure 1:**
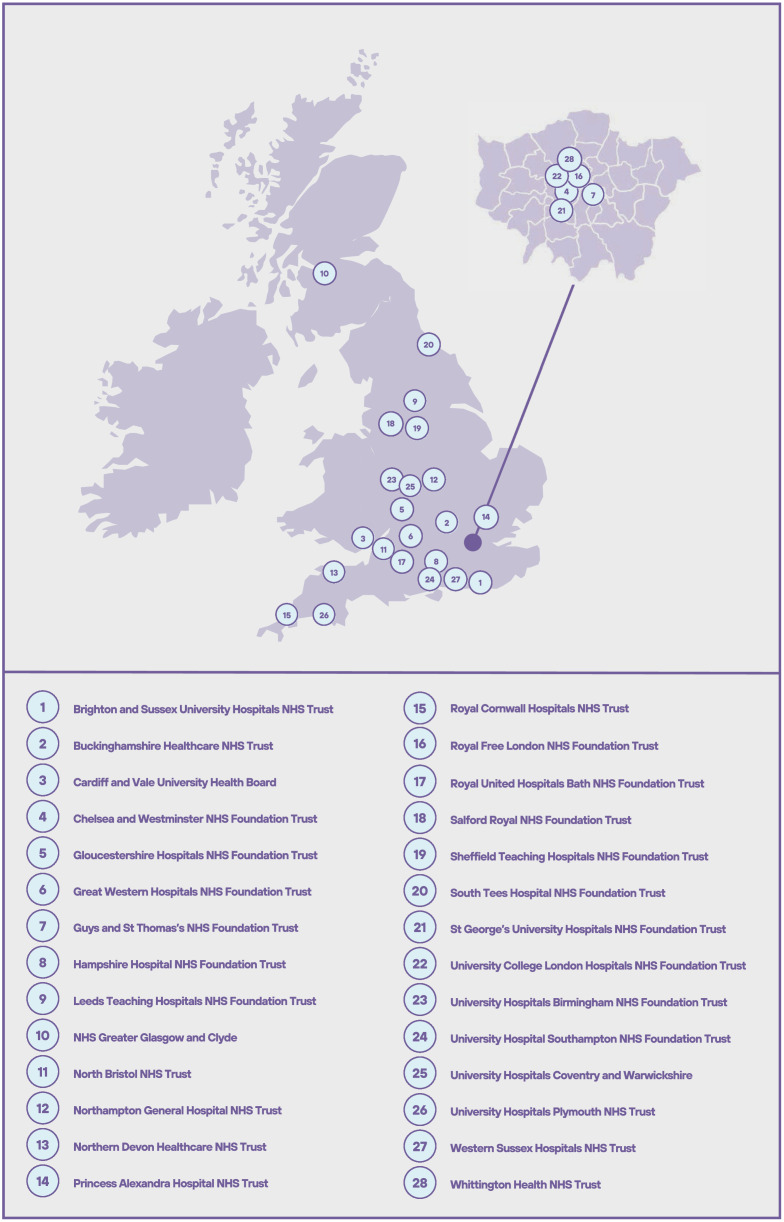
Infographic displaying the geographical locations of the recruiting audit centres.

Volunteer consultant orthopaedic, plastic or hand surgeons who were willing to undertake an evaluation of preventable hand injuries over a two-week period from 00.01 hrs on Monday 22 February 2021 to 24:00 hrs on Sunday 7 March 2021(Supplementary Table 1) were sought throughout the UK. The participating hospitals (n=28) represent a mix of district general hospitals, major trauma centres and specialist academic and teaching institutions (Figure 1). Each hospital recorded the injuries of all consecutive patients presenting to their department over the two-week period. The inclusion criteria were patients with hand injury requiring a referral to a secondary care orthopaedic, plastic or hand surgical team. The exclusion criteria were patients whose injuries were sufficiently mild and did not require a referral to surgery, such as a superficial laceration with no deeper injury or a soft tissue injury that did not require specialist intervention after emergency department (ED) assessment.

### Population

Data including patient demographics, types and mechanisms of injury as well details of whether the patient admitted to being intoxicated or under the influence of illegal drugs at the time of presentation to the ED were collected. Preventable injury criteria were decided *a priori* (GG, MH, JL). Specifically, it was considered that injuries associated with sport were not preventable, as there are inherent risks to playing sport but the benefits of playing sport outweigh the risks involved. Moreover, boxer's fractures were considered not preventable without change in human behaviour. These data were entered into a spreadsheet with drop down menus to optimise accuracy and a descriptor column in case of doubt. The descriptors of the injury mechanisms were assessed (ALW, MH, GG, JL) to evaluate whether they were preventable or not; when in doubt, they were considered to be not preventable. The cost of each individual treatment could not be assessed in detail; however, data on the treatments provided an indication of the costs involved.

### Statistical methods

Data was descriptively analysed only due to the nature of study design.

## Results

Data were available from 28 hospitals representing a reasonable geographical spread throughout the UK. In total, 1909 patients were eligible for assessment, including 184 children under 16 years of age. There were 1118 men, and 789 women; and in two patients, the sex was not recorded. There were slightly more injuries on the right side (right 955 vs left 919) with bilateral injuries in 35 patients. The median age of all patients was 40 years (interquartile range [IQR, 25-59 years]); the median age of the adults (≥ 16 years) was 44 years (IQR, 29-61 years); the median age of the children was 10 years (IQR, 6-12 years). The median time from injury to attendance in the ED or at their general practice surgery (GP) practice was 0 days (same day; IQR, 0-1 day). The median time from attendance in the ED or at their GP practice for review by a specialist was 1 day (IQR, 0-3 days).

The five most commonly recorded injuries were fractures of the wrist, single phalangeal or metacarpal fractures, fingertip injuries and infections (Table 1). However, 175 patients (9.1%) could not be assigned any injury classification.

**Table 1:** Injuries presented in order of frequency.

Main Injury Classification	Frequency (N)	%*
Single phalanx fracture	258	15
Wrist fracture	254	15
Single metacarpal fracture	221	13
Fingertip injury	170	9.8
Infection	133	7.7
Carpus fracture	96	5.5
Complex or multiple Injury	93	5.4
Tendon - extensor Single	92	5.3
Nailbed injury	92	5.3
Small joint or carpus dislocation	51	2.9
Volar plate	51	2.9
Nerve injury	50	2.9
Combination of injuries	45	2.6
Multiple MC fracture	35	2.0
Tendon - flexor single	29	1.7
Multiple phalanx fracture	17	1.0
Tendon - flexor multiple	17	1.0
Soft tissue injury	8	0.5
Tendon - extensor multiple	9	0.5
Other	8	0.5
Ulnar or radial collateral ligament injury	5	0.3
Total	1734	

⁎ percentage of injuries where injury known: total 1734.

The mechanisms of injury are shown in Table 2, highlights the differences between preventable and non-preventable injuries. The recorded mechanism of injury was unavailable for 37 patients (1.9%), with mechanical falls and manual labour being the commonest causes of injury. Fifty percent of injuries were considered preventable. Overall, the association of injuries with drugs or alcohol use was limited, with only 37 (1.9%) cases reported as being associated with alcohol intake and 8 (0.54%) with drug use.

**Table 2:** Top mechanisms of injury.

Mechanism	Frequency (N)	Percent (%)	Preventable?
Mechanical fall - outdoors	308	16	Not preventable
Mechanical fall at home	229	12	Preventable
Manual labour	137	7.2	Preventable
Mechanical fall during sport / exercise	108	5.7	Not preventable
DIY related injury	156	8.2	Preventable
Door/window	98	5.1	Preventable
Kitchen/cooking	128	6.7	Preventable
Punch Injury - wall/glass/other object	96	5.0	Not Preventable
Sports injury	85	4.5	Not preventable
Animal bite	84	4.4	Not preventable
Machine related injury - other	80	4.2	Preventable
Assault victim - other	58	3.0	Not Preventable
RTA- bicycle/scooter	52	2.7	Not preventable
Glass cut	49	2.6	Preventable
Machine related injury - crush	52	2.7	Preventable
RTA - bike/car	35	1.8	Not preventable
Punch injury - assault	28	1.5	Not Preventable
Ring entrapment	23	1.2	Preventable
Deliberate self-harm	10	0.5	Not Preventable
Knife injuries	7	0.4	Preventable
Assault victim - fight bite	4	0.2	Not Preventable
Other	45	2.4	NA
Unknown	20	1.0	NA
Missing data	17	0.9	NA
	1909		

## Discussion

### Principle findings

This study demonstrates that it is possible to describe preventable injuries in patients presenting to hospitals in the UK. Furthermore, it was found that a large number of preventable and non-preventable hand injuries are presented to hand specialists in the UK. However, the data were collected during a period of national lockdown secondary to the COVID-19 pandemic. Therefore, the results are likely to be an underestimate of the true burden of injuries during “normal, non-pandemic” socialising situations. This study was planned as a pilot prior to a definitive study to compare injuries during a national lockdown to a period of relatively normal activity.

Data were collected from 28 hospitals covering an estimated catchment totalling over 14 million people. Considering this sample to be representative and extrapolating this to the rest of the UK population, we estimated that approximately 4500 potentially preventable injuries occurred each week, totalling over 230,000 per year.

Furthermore, a high proportion of fractures compared to soft tissue injuries were observed. In general, these injuries often require multiple hospital visits and specialist input from both surgeons and therapists.^7^ Seven injuries that were treated non-operatively mostly required only one appointment with a specialist surgeon, but represented an additional burden to the hand therapy services. Injuries requiring surgery will incur greater healthcare costs. Although no formal cost analysis was performed, these potentially preventable hand and wrist injuries represent a substantial financial burden to society, which potentially could be mitigated by using an injury prevention strategy. Such a strategy would likely have benefits extending to the wider society in terms of reduced absence from work or caring roles. De Putter *et al.* estimated that the medical and societal cost of hand injuries each year was $740 million in the Netherlands.^8^ However, their population is 17 million compared to 67 million in the UK. This suggests a yearly cost of $2920 million for hand injuries in the UK. Based on a preventable injury rate of 50%, one could estimate that reducing preventable injuries by as little as 10% could potentially save approximately $146 million. Accurate estimates of costs and potential savings are not possible within the data collected here; however, future studies could focus on identifying the remuneration codes used for hospital associated episodes.

Men were considerably more likely than women to suffer injuries in general and preventable injuries in particular. The most common location for preventable injuries was at home, but injuries also occurred at work, despite the national lockdown. We considered that almost all injuries at work are preventable. At home, the preventable injuries were primarily associated with mechanical falls and do it yourself, in addition to injuries around doors and those associated with kitchen activities. Extensive work has been undertaken historically to address safety issues at the workplace, but high level reviews note a lack of evidence for several recommendations provided in the health and safety measures.^9^^,^^10^ At home, it is likely that injuries are caused through impatience, inexperience and several individual mechanisms, including taking the pips out of avocados and separating frozen bread or hamburgers with a sharp knife, for hand injuries have previously been reported.^11^^,^^12^ Moreover, research focussed on adaptation of the home environment to prevent injury has generated mixed results.^13^

### Limitations and future work

There are limitations to this study. This study was undertaken during the COVID lockdown, and in a less unusual time the data generated may have been different. Inevitably, not every patient would have been accounted for; some may have been referred out of the catchment area (likely to be balanced by those referred in); and some patients may have been missed. Additionally, not all the data were complete. Another particular limitation is that the assessment of preventable and non-preventable injuries is biased by subjectivity. Efforts were made to limit subjectivity via consensus agreement between three individuals. The costs were not formally analysed, meaning that only estimates were made based on previous studies.^8^

Furthermore, defining preventable and non-preventable injuries within a category based on mechanism rather than a free text analysis on a case-by-case basis can be considered as another limitation. This definition method was designed to be pragmatic, encouraging clinical collaborators at multiple sites to engage in data collection. We felt that through an iterative process we could establish a ‘best case’ definition of what was or was not preventable, which could be suitably used with this study design. As a multicentre study that required data sharing, data had to be anonymised and classified into injury groups prior to being sent to a central study team for analysis. This prevented secondary disclosure of data, but this also meant that some granular information was lost. This granular data would enable more specific definition on a case-by-case basis. We did consider further case definition at each site, but the potential for case definition variation between sites and low interobserver reliability was considered significant enough to introduce bias. When formulating study design, we felt that the external validation throughout a wide geographically and socioeconomically mixed area, along with a greater sample size was worth the loss of granularity at each site. This method enabled us to obtain the most complete data possible at each of the 28 sites, and aided in the design of a replicable method of descriptive analysis of data. A limitation of this technique, however, is the potential loss of the nuance of injury mechanism, which could lead to misclassification.

We must also acknowledge that the data was collected during a unique period, where communal activities such as contact sports did not take place due to lockdown restrictions. Whilst this makes the data associated with the pandemic interesting, a lower than anticipated proportion of preventable injuries may have been presented. Future work is needed to compare data collected during this period to a comparable period without restrictions in place. This is a multicentre audit, but it should be complimented by other studies focussing on smaller study areas that offer greater granularity of information.

This is the first extensive survey of preventable hand injuries and shows how common they are in a representative sample of 28 NHS trusts (approximately 12% of all trusts) in the UK. Preventable hand injuries are common and potentially expensive. Investing in the prevention of injuries would appear worthwhile.

## Ethical Approval

The study was approved at each contributing site with local research governance approval prior to data collection.

## Contributor Statement

GG and MH conceived the study and GG, MH and JCEL designed the study method. All collaborators and ALW acquired the data. JCEL performed the data analysis, with GG, MG and JCEL interpreting the data. JCEL, MH and GG drafted the final manuscript. All authors were involved in the acquisition of data and in drafting, reviewing, and editing the final manuscript. JCEL, MH, GG, and ALW had full access to all data in this study. GG and MH are guarantors.

## Role of Funding Source

This work acknowledges the support of the National Institute for Health and Care Research Barts Biomedical Research Centre (NIHR203330); a delivery partnership of Barts Health NHS Trust, Queen Mary University of London, St George's University Hospitals NHS Foundation Trust, and St George's University of London.

The funding source had no role in the study design, collection, analysis, interpretation of data, writing of the manuscript, or decision to submit for publication.

## Data Statement

Further data may be available upon written request to the authors, noting that aggregated data will only be released to prevent secondary disclosure of data from the pseudonymised dataset.

## Conflict of Interest Statement

All authors have completed an ICJME conflict of interest form that is uploaded with the article; (http://www.icmje.org/conflicts-ofinterest/) no support was received from any organization for this study.

## Appendix Supplementary materials

Image, application 1
