# Supplementary material for: Preventable hand injuries: A national audit
Source: JPRAS Open. 2023 Oct 5;38:305–12. doi: 10.1016/j.jpra.2023.10.005 (PMC10684376; doi:10.1016/j.jpra.2023.10.005)
Supplement: Supplementary file 1 [file mmc1.docx]

**Supplementary Table 1.** Participating acute hospital trusts

| **Location** | **Catchment (2019)** |
| --- | --- |
| Gloucestershire Hospitals NHS Foundation Trust | 587135 |
| Great Western Hospitals NHS Foundation Trust | 354829 |
| Northern Devon Healthcare NHS Trust | 155294 |
| University Hospitals Plymouth NHS Trust | 478657 |
| Hampshire Hospital NHS Foundation trust | 441864 |
| Cardiff and Vale University Health Board | 329500 |
| University Hospitals Coventry and Warwickshire | 586037 |
| Salford Royal NHS Foundation Trust | 296390 |
| Royal Free London NHS Foundation Trust | 692640 |
| Chelsea & Westminster NHS Foundation Trust | 611653 |
| Guy’s’ and St Thomas’ NHS Foundation Trust | 858712 |
| St George’s University Hospitals NHS Foundation Trust | 569705 |
| University College London Hospitals NHS Foundation Trust | 617520 |
| Sheffield Teaching Hospitals NHS Foundation Trust | 691174 |
| University Hospital Birmingham NHS Foundation Trust | 1174270 |
| NHS Greater Glasgow and Clyde | 320000 |
| Buckinghamshire Healthcare NHS Trust | 402331 |
| Princess Alexandra Hospital NHS Trust | 250690 |
| Royal Cornwall Hospitals NHS Trust | 437694 |
| Northampton General Hospital NHS Trust | 354159 |
| University Hospital Southampton NHS Foundation Trust | 639804 |
| Whittington Health NHS trust | 202126 |
| North Bristol NHS Trust | 514093 |
| Leeds Teaching Hospitals NHS Trust | 865127 |
| Brighton and Sussex University Hospitals NHS Trust | 528111 |
| South Tees Hospital NHS Foundation Trust | 488804 |
| Royal United Hospital Bath NHS Foundation Trust | 407976 |
| Western Sussex Hospitals NHS Trust | 441218 |
